# Cranial Irradiation Alters Dendritic Spine Density and Morphology in the Hippocampus

**DOI:** 10.1371/journal.pone.0040844

**Published:** 2012-07-16

**Authors:** Ayanabha Chakraborti, Antino Allen, Barrett Allen, Susanna Rosi, John R. Fike

**Affiliations:** 1 Department of Neurological Surgery, University of California San Francisco, San Francisco, California, United States of America; 2 Department of Radiation Oncology, University of California San Francisco, San Francisco, California, United States of America; 3 Department of Physical Therapy and Rehabilitation Science, University of California San Francisco, San Francisco, California, United States of America; 4 Brain and Spinal Injury Center, University of California San Francisco, San Francisco, California, United States of America; National Cancer Institute, United States of America

## Abstract

Therapeutic irradiation of the brain is a common treatment modality for brain tumors, but can lead to impairment of cognitive function. Dendritic spines are sites of excitatory synaptic transmission and changes in spine structure and number are thought to represent a morphological correlate of altered brain functions associated with hippocampal dependent learning and memory. To gain some insight into the temporal and sub region specific cellular changes in the hippocampus following brain irradiation, we investigated the effects of 10 Gy cranial irradiation on dendritic spines in young adult mice. One week or 1 month post irradiation, changes in spine density and morphology in dentate gyrus (DG) granule and CA1 pyramidal neurons were quantified using Golgi staining. Our results showed that in the DG, there were significant reductions in spine density at both 1 week (11.9%) and 1 month (26.9%) after irradiation. In contrast, in the basal dendrites of CA1 pyramidal neurons, irradiation resulted in a significant reduction (18.7%) in spine density only at 1 week post irradiation. Analysis of spine morphology showed that irradiation led to significant decreases in the proportion of mushroom spines at both time points in the DG as well as CA1 basal dendrites. The proportions of stubby spines were significantly increased in both the areas at 1 month post irradiation. Irradiation did not alter spine density in the CA1 apical dendrites, but there were significant changes in the proportion of thin and mushroom spines at both time points post irradiation. Although the mechanisms involved are not clear, these findings are the first to show that brain irradiation of young adult animals leads to alterations in dendritic spine density and morphology in the hippocampus in a time dependent and region specific manner.

## Introduction

Cranial irradiation is an essential therapeutic tool in the treatment of primary and secondary malignancies, but can be associated with a risk for adverse side effects, including cognitive dysfunction [1] which can severely affect quality of life [2]. Currently there are no successful long-term treatments or preventive strategies for radiation-induced cognitive impairments [3]. Thus, a better understanding of the cellular and molecular factors that may lead to the development of such changes is essential for the management of this serious complication and for designing effective therapeutic strategies.

The hippocampus plays a crucial role in learning and memory [4] and considerable data exist showing that irradiation leads to impairment of those functions [5]–[7]. This structure is composed of anatomically distinct but functionally interrelated subfields consisting of different cell types, cell sizes, neural connectivity, electrophysiological properties and susceptibility to insult [8]. The dentate gyrus (DG) is one of the two brain regions where neurogenesis takes place throughout life [9] and has been shown to be particularly susceptible to radiation [10], [11]. In contrast, neural degeneration and loss associated with Alzheimer’s disease, epilepsy or ischemic/anoxic episodes are seen more distinctively in the CA1 region than in any other brain area [12]. There have also been reports suggesting differences in responses between the CA1 pyramidal cells and DG granule cells after given injurious stimulus [13], but there is a paucity of information regarding sub region specificity in the effects of irradiation on the hippocampus.

The formation of long-term memory relies on modulation of synaptic connections in response to neuronal input. This plasticity requires coordinated activity-dependent synthesis of specific mRNAs and proteins that facilitate molecular and structural changes at the synapse [14]. Dendritic spines are bulbous membrane projections that form the postsynaptic specializations of the vast majority of excitatory synapses in the central nervous system (CNS) and their structure and density are important factors in synaptic function [15]. Spines exhibit a variety of shapes and sizes and are generally categorized into thin (long neck and small head), mushroom (well defined neck and very voluminous head) and stubby (no neck and stubby appearance) types [16], [17]. Spine morphology can predict both spine stability and synaptic strength, as large spines tend to form strong synapses and small spines are generally transient and form weaker synapses [18], [19]. Changes in dendritic spine density or structural reorganization of spines is thought to be important for cognitive processes such as learning and memory and dendritic spine remodeling has been correlated with changes in the strength of excitatory synaptic transmission [20]. Several neurological and psychiatric disorders exhibit abnormal dendritic structure and/or alterations in dendritic spine morphology [21]. However, little is known about the potential effects of brain irradiation on dendritic spines in the hippocampus in young adult animals. A better knowledge of how cranial irradiation affects dendritic spines in hippocampal sub regions could provide critical information regarding the mechanism of disruption of neural circuitry following radiation exposure.

The purpose of the present study was to determine the temporal effects of cranial irradiation on spine density and morphology in the dendrites of granule neurons of dentate gyrus as well as pyramidal neurons of CA1 area of the hippocampus. Since pyramidal neurons typically consist of apical and basal dendrites which differ in their connectivities, biophysical characteristics and long term potentiation induction and expression mechanisms [22], [23], spine analyses were conducted separately in the apical and basal dendrites. To the best of our knowledge, no previous experiments have specifically addressed temporal and region specific effects of cranial irradiation on spine density and morphology in the hippocampus in young adult animals. Therefore, this was designed as a proof of concept study using a dose of irradiation that has been shown to cause hippocampal dependent cognitive impairment, so as to determine if changes in dendritic spines might offer a specific target for better understanding the effects of irradiation on cognition.

## Methods

### Ethics Statement

The study was carried out in strict accordance with the recommendations in the Guide for the Care and Use of Laboratory Animals of the National Institutes of Health. The protocol was approved by the University of California, San Francisco (UCSF) Institutional Animal Care and Use Committee (IACUC).

A total of 20 two month old male C57BL/6J mice (n = 5/group) were used for this study. Animals were purchased from a commercial vendor (Jackson Laboratory, Bar Harbor, ME) and were group housed (5 mice/cage) throughout the study. Mice were maintained on a 12-h light-dark cycle and were provided food and water ad libitum. All efforts were made to minimize suffering of the animals.

### Irradiation

For irradiation, mice were anesthetized using an i.p injection of ketamine (70 mg/kg) and medetomidine (0.5 mg/kg). Mice were placed prone, 16.3 cm from a cesium-137 source (J.L. Shepherd & Associates, San Fernando, CA) and shielded with an iron collimator that limited the irradiation to a 1 cm wide vertical beam [24]. The beam entered laterally, and to insure a uniform dose across the entire brain, half the dose was given from each side of the head. A single dose of 10 Gy was used because previous studies have shown that cognitive impairments, decreases in neurogenesis and decreases in the expression of the plasticity-related immediate early gene *Arc* were induced by this dose [25]–[27]. The total time to deliver 10 Gy was approximately 6 minutes. Unirradiated mice were anesthetized similarly to those that were irradiated. One week or 1 month post irradiation the animals were euthanized by cervical dislocation.

### Golgi Staining

For spine analyses, Golgi staining was performed using the FD Rapid Golgi Stain Kit (FD Neurotechnologies, Baltimore, MD) [28], [29], following the manufacturer’s guidelines. Briefly, freshly removed brains were immersed in a proprietary impregnation solution and stored at room temperature for 2 weeks in the dark. Next, the brains were transferred to a second impregnation solution and incubated for 48 hours at 4°C. Finally, the tissues was shipped to FD Neurotechnologies, where they were sectioned to a thickness of 120 µm, stained and then mounted on gelatin coated slides. The slides were sent back to UCSF for microscopic analyses.

### Analysis of Dendritic Spine Density and Spine Morphology

**Figure 1:**
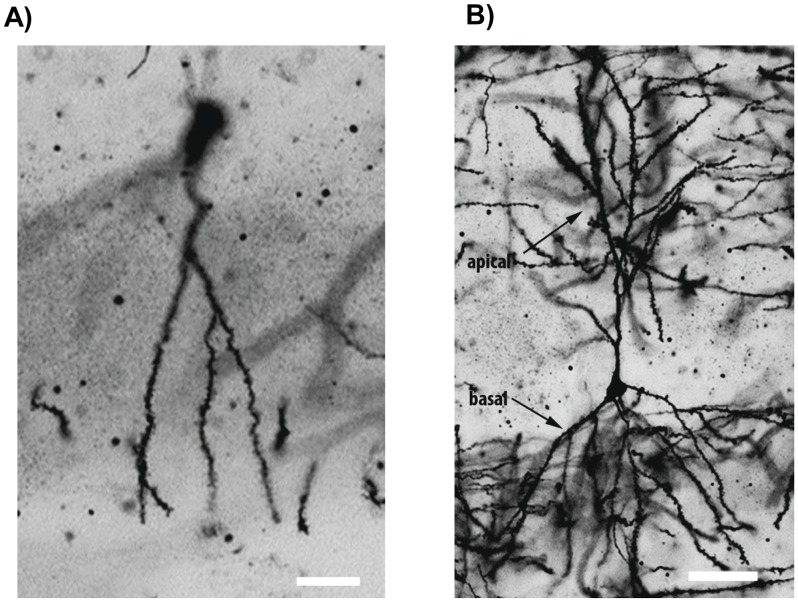
Representative images of Golgi-impregnated cells in the mouse hippocampus. (A) A dentate gyrus granule neuron and (B) a CA1 pyramidal neuron illustrating basal dendrites in the stratum oriens and apical dendrites in the stratum radiatum. Scale bar = 50 µm.

Spine analyses were conducted blind to the experimental conditions on coded Golgi impregnated brain sections containing the dorsal hippocampus. Spines were examined on dendrites of DG granule neurons (**Fig. 1A**) as well as apical (stratum radiatum) and basal (stratum oriens) dendrites of CA1 pyramidal neurons (**Fig. 1B**).

The neurons that satisfied the following criteria were chosen for analysis in each of the experimental groups: i) presence of untruncated dendrites; ii) consistent and dark Golgi staining along the entire extent of the dendrites; and iii) relative isolation from neighboring neurons to avoid interference with analysis [30]. Three-5 dendritic segments, each at least 15 µm in length [31], were analyzed per neuron, and 10–11 neurons were analyzed per brain.

**Figure 2:**
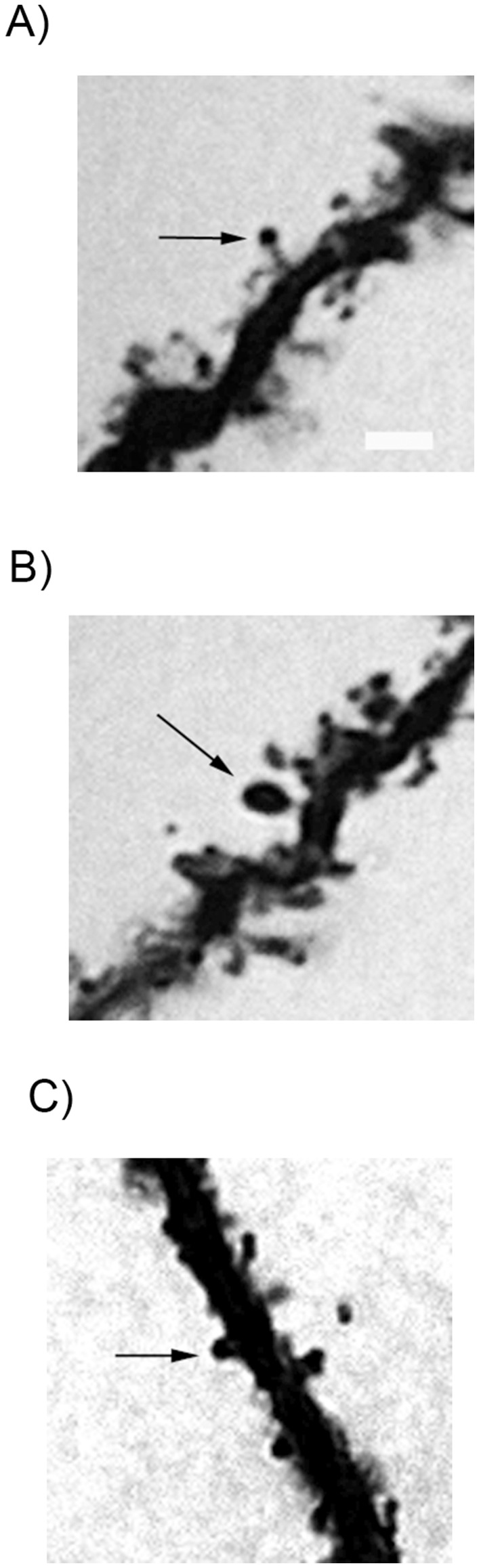
Representative images of spine morphological sub types. (A) Thin (B) Mushroom and (C) Stubby spines (arrows). Scale bar = 5 µm.

On the basis of morphology, spines were classified into the following categories: i) Thin: spines with a long neck and a visible small head; ii) Mushroom: big spines with a well-defined neck and a very voluminous head; and iii) Stubby: very short spines without a distinguishable neck and stubby appearance [16], [17], [31] (**Fig. 2**).

To acquire images for spine analysis, the dendritic segments were imaged under brightfield illumination on a Zeiss Axioimager microscope with a 63x oil immersion objective. Spine analyses were based on the method of Margarinos et al [31]. This method does not assess spine density in a 3 dimensional manner but focuses on spines that are parallel to the plane of section. Although the method may underestimate the the total number of spines, it facilitates a direct comparison of treatment groups when they are analyzed in an identical manner [31]. Image J software was used to calculate linear spine density [32], which was presented as the number of spines per 10 µm of dendrite length.

### Statistical Analysis

Data were expressed as Mean ± SEM. An unpaired two-tailed *t*-test with Welch’s correction was used to evaluate statistical differences between sham and irradiated groups. All statistical analyses were conducted using GraphPad Prism 5.0 software (La Jolla,CA), and p≤0.5 was considered significant.

## Results

### Changes in Dendritic Spine Density after Radiation Exposure

**Figure 3:**
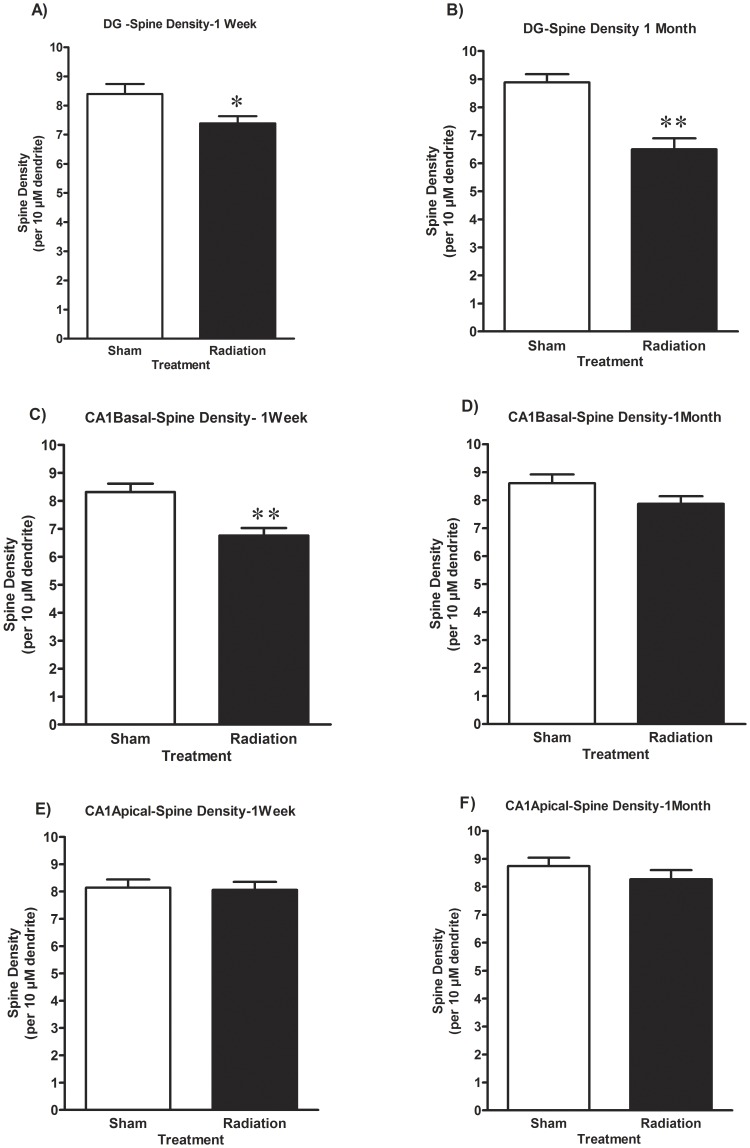
Irradiation affects dendritic spine density in the hippocampal DG and CA1 area. Brain irradiation induced time and region specific changes in the numbers of dendritic spines/10 µm in the DG granule neurons as well as pyramidal neurons in the CA1 region. The open bars represent unirradiated animals and the dark bars represent mice irradiated with 10 Gy of gamma rays. Each bar represents the mean value of 5 animals and error bars are SEM. *represents a p<0.05, and **represents p<0.001.

In the DG, there were significant reductions in spine density at both 1 week (11.9%, p<0.05) and 1 month (26.9%, p<0.001) after irradiation (**Fig. 3A & B**). In contrast, in the basal dendrites of CA1 pyramidal neurons, irradiation resulted in a significant reduction (18.7%, p<0.001) in spine density only at 1 week post irradiation while changes observed at 1 month were not statistically significant (**Fig. 3C & D**). Irradiation did not significantly alter spine density in the CA1 apical dendrites at either time after irradiation (**Fig. 3E & F**).

### Changes in Dendritic Spine Morphology after Radiation Exposure

**Figure 4:**
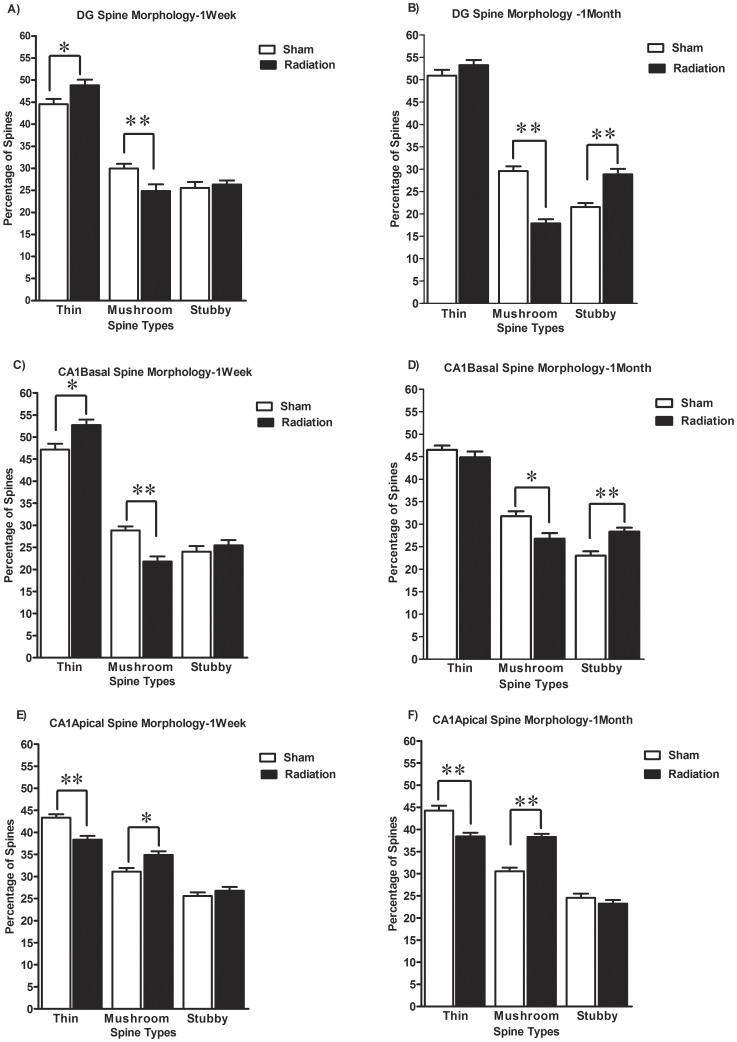
Irradiation affects dendritic spine morphology in the hippocampal DG and CA1 area. Brain irradiation induced time and region specific changes in the proportion of spine morphological sub types in the DG granule neurons as well as pyramidal neurons from the CA1 region. The open bars represent unirradiated animals and the dark bars represent mice irradiated with 10 Gy of gamma rays. Each bar represents the mean value of 5 animals and error bars are SEM. *represents a p<0.05, and **represents p<0.001.

In the DG, 1 week after irradiation, there was a significant increase (9.6%, p<0.05) in the proportion of thin spines while at 1 month the difference was not statistically significant (**Fig. 4A & B**). On the other hand, the proportion of mushroom spines was significantly reduced at both 1 week (17%, p<0.05) and 1 month (39.5%, p<0.001) after irradiation (**Fig. 4A & B** **)**. The proportion of stubby spines was significantly increased (34.1%, p<0.001) only at 1 month post irradiation while the changes observed earlier were not statistically significant (**Fig. 4A & B** **)**.

In the CA1 basal dendrites, irradiation significantly increased the proportion of thin spines at 1 week (11.7%, p<0.05) but no changes were observed at the later time point (**Fig. 4C & D**). On the other hand, significant reductions in the proportion of mushroom spines were observed at both 1 week (24.3%, p<0.001) and 1 month (15.7%, p<0.05) post irradiation (**Fig. 4C & D**). Although no significant differences were observed in the proportion of stubby spines between sham and irradiated group at 1 week, there were significant differences (23.1%, p<0.001) in the proportion of this spine subtype at 1month (**Fig. 4C & D**).

Despite the fact that no changes in spine density were observed in the CA1 apical dendrites at either time point, there were significant differences in both thin and mushroom spine morphology between the sham and irradiated groups (**Fig. 4E & F**). Contrary to what was observed in DG and CA1 basal dendrites, there was a significant decrease in the proportion of thin spines at both 1 week (11.49%, p<0.001) and 1 month (13.2%, p<0.001), as well a significant increase in proportion of mushroom spines at 1 week (12.1%, p<0.05) and 1 month (25.3%, p<0.001) post irradiation (**Fig. 4E & F**). No significant differences were observed in the proportion of stubby spines between sham and irradiated group at either 1week or 1 month post irradiation (**Fig. 4E & F**).

## Discussion

The present study demonstrated that brain irradiation altered spine density as well as the proportion of morphological subtypes in the dendrites of DG granule neurons and basal dendrites of CA1 pyramidal neurons in a time dependent manner. While there was a gradual decrease in spine density in the DG over time, spine density in the CA1 basal dendrites decreased at 1 week post irradiation with a trend toward recovery at 1 month. Additionally, in the CA1 apical dendrites, irradiation altered spine morphology without any change in spine density at both 1week and 1month post irradiation. To our knowledge, these results are the first to demonstrate that, in young adult mice, cranial irradiation affects dendritic spine density and morphology in the hippocampus in a temporal and region specific manner.

The maintenance of normal brain function is dependent on the establishment and efficient maturation of synaptic circuits [33]. The hippocampus plays a key role in learning and memory processes [34] and is particularly susceptible to the effects of ionizing irradiation [10], [11]. While irradiation has been shown to change the numbers of newly born neurons in the DG [25], data also exist showing changes associated with learning and memory that do not involve overt mature neuronal loss [27]. This latter finding suggests that changes in structure and function of viable neuronal cells may play an important role in the development of cognitive deficits after irradiation, and highlights the potential importance of assessing critical structures such as dendritic spines.

Dendritic spines are the primary recipients of excitatory input in the CNS, and changes in spine density and morphology can account for functional differences at the synaptic level [35]. Spine morphology can predict both spine stability and synaptic strength [18] and findings from *in vivo* models support the notion that structural plasticity of spines is related to learning and memory function [36], [37]. Spines also compartmentalize Ca^2+^ and other signaling components conferring specificity to changes in synaptic efficacy and protecting neurons from excitotoxicity [38]. In light of the multiple spine functions, pathological changes in spine number and structure may have significant consequences for brain function, as has been shown in studies of stress, malnutrition, toxins and drugs of abuse [39]–[42].

Golgi staining is a reliable and sensitive method for revealing the morphological details of individual neurons, especially dendritic spines [29]. One drawback to this technique is that it cannot be effectively combined with other staining techniques [43]. Because the goal of the present study was only to address spine density and morphology and not to identify other cell types, we selected this method over other available techniques for spine analysis. The analysis of Golgi stained neurons showed that radiation exposure led to a gradual decrease in spine density in the DG over time. In contrast, spine density in the CA1 basal dendrites decreased at 1 week post irradiation with a trend toward recovery at 1 month. The observed reductions in spine density might indicate early signs of neuronal injury in the hippocampus following irradiation and also suggest that there is a time dependent vulnerability of the two hippocampal sub regions following radiation exposure.

A number of factors might account for the observed differences in spine density between the two hippocampal subregions. Numerous studies have demonstrated that spine density is regulated by glutamatergic transmission and glutamate receptor subtypes located on dendritic spine heads [44]–[46]. In addition, a series of *in vitro* studies have shown that N-methyl-D-aspartic acid (NMDA) receptors mediate the destabilization of filamentous actin (f-actin) associated with dendritic spine loss [46], [47]. Although the effects of radiation on NMDA receptor dynamics on hippocampal sub-regions are not well understood, studies by Shi et al [48] have shown differential changes in subunits of NMDA receptors in the hippocampal subfields following whole brain irradiation. Thus, it is tempting to speculate that the observed temporal differences in reduction in spine density between DG and CA1 basal dendrites may involve differential alterations of NMDA receptor mediated responses in these two areas following irradiation. Brain derived neurotrophic factor (BDNF) is another well characterized determinant of dendritic spine number and morphology [49]. Regulation of BDNF and its receptor expression has been reported to be very sensitive to radiation in the hippocampus and such changes vary depending on time after irradiation [50]. Therefore, it is also possible that radiation might differentially alter BDNF and its downstream signaling targets in the dendrites of dentate granule cells and CA1 basal dendrites which may account for the differential changes in spine density at these two regions as a function of time after irradiation.

In our earlier studies using the same dose of radiation in the same strain of mice, we found increased numbers of activated microglia in the DG 1 week, which became significant at 2 months post irradiation [27]. Therefore, the gradual decrease in spine density over time observed in the DG (**Fig. 3**) could be associated with an increase in microglial activation. Other investigators have recently shown that changes in dendritic spines are associated with alterations in microglia [51], an effect that may be associated with the release of soluble factors [52]. Further studies are in progress to address the molecular mechanisms involved in the observed temporal differences in radiation induced alterations in spine density.

In contrast to DG and CA1 basal dendrites, irradiation did not alter spine density in CA1 apical dendrites. Differential vulnerability between basal and apical dendrites due to exogenous or endogenous factors has been reported in the literature although the mechanisms involved are not clear. For instance, Santos et al [53] reported that neonatal rats exposed repetitively to low doses of paroxon (a organophosphate-type cholinesterase inhibitor) lost dendritic spine selectively in basal dendrites with no changes in apical dendrites of CA1 pyramidal neurons. Moreover normal aging also results in decreases of the spine density on basal but not apical dendrites in C57BL/6 mice [54]. Future studies will be required to evaluate the mechanistic basis of differential vulnerability in radiation induced reduction of spine density between CA1 basal and apical dendrites.

One of the most remarkable features of dendritic spines is their morphological diversity [55]. The three categories studied here appear to have different functional properties, including activity induced changes in intracellular calcium concentration, glutamate receptor levels and perhaps new versus well established memory processing [56]. Additionally, dendritic spine morphology has also been reported to affect the diffusion and compartmentalization of membrane associated proteins and expression of α-amino-3-hydroxy-5-methyl-4-isoxazolepropionic acid (AMPA) receptors [57]. Given this information, we assessed whether the proportions of each type of spine were altered in the DG and CA1 area following radiation exposure.

Our data showed that in both DG and CA1 basal dendrites, spines characterized by the mushroom morphology were particularly affected by radiation exposure. Mushroom spines have larger postsynaptic densities [17] which anchor more AMPA glutamate receptors and make these synapses functionally stronger [58]. Mushroom spines are more likely to contain smooth endoplasmic reticulum, which can regulate calcium locally [59] and spines that have larger synapses are also more likely to contain polyribosomes for local protein synthesis [60], [61]. Thus, the loss of mushroom spines as seen here may have a more profound effect on neuronal function than the loss of the other types of spines. Gao *et al* [29] has also recently reported that moderate traumatic brain injury in mice led to significant decrease in mushroom shaped spines indicating a reduction in number of synapses which was confirmed by synaptophysin staining.

Whereas radiation exposure led to decrease in the fraction of mushroom spines, a marked increase in the proportion of stubby spines were observed in both DG and CA1 basal dendrites 1 month post irradiation. Although less is known about these stubby structures, they have been shown to predominate early in postnatal development [62] and to increase in mature hippocampal slices after synaptic transmission was blocked [63]. It has also been reported that dopamine receptors are located on the spine neck in the perisynaptic space [64] and stubby spines that lack a neck likely have abnormal distributions of dopamine receptors in this space [65]. It can be speculated that a marked increase in the proportion of stubby spines by radiation exposure might therefore lead to some alterations in dopaminergic signaling. Because radiation has been reported to affect dopaminergic processes in the brain [66], such changes may have long-term consequences for radiation induced cognitive changes.

Despite the fact that no change in spine density was observed in the apical dendrites of CA1 neurons after irradiation, significant differences in thin and mushroom spine morphology were observed between the sham and irradiated groups. It is noteworthy that contrary to what was observed in DG and CA1 basal dendrites, irradiation led to significant decreases in the percentages of thin spines after irradiation and a significant increase in mushroom spines. The length of the spine neck seems to be a key regulator of spinodendritic Ca^2+^ signaling and of the transmission of membrane potentials [67]. Thin spines maintain the structural flexibility to enlarge and stabilize after long term potentiation and can accommodate new, enhanced or recently weakened inputs, making them candidate ‘learning spines’ [14], [68]. By decreasing the proportion of learning spines, radiation may therefore decrease a neuron’s ability to form new synapses and changes in activity in the CA1 apical dendrites. Age related reductions in thin spines have been observed in rhesus monkeys, with cognitive performance inversely proportional to thin spine volume [69]. Although the reason for the corresponding increase in mushroom spines in these dendrites is not clear, it might represent a homeostatic mechanism to compensate for the reduction of the learning spines.

The functional implications of the observed radiation effects on dendritic spines at the two hippocampal sub regions are not yet clear. Additionally, if or how these radiation-induced alterations may relate to the behavioral [25], [26], cellular [10], [70] and *Arc* changes [27] observed at the same dose and/or time used here, remains to be determined.

In conclusion, to the best of our knowledge the present report provides the first evidence that in young adult mice, cranial irradiation causes alteration in spine density and morphology in the hippocampus in a time dependent and region specific manner. Since loss of dendritic spines or structural reorganizations of spines play an important role in learning and memory, the observed changes suggest a disruption of neural circuitry that might play a role in radiation induced cognitive impairment.
